# Supplementary material for: Cost-effectiveness analysis of romosozumab for severe postmenopausal osteoporosis at very high risk of fracture in Mexico
Source: PLoS One. 2025 Feb 7;20(2):e0299673. doi: 10.1371/journal.pone.0299673 (PMC11805434; doi:10.1371/journal.pone.0299673)
Supplement: S1 Table — (DOCX) [file pone.0299673.s001.docx]

**S1 Table General population annual fracture rates**

| **Age (years)** | **Hip fracture** | **Vertebral fracture** | **Other fracture** |
| --- | --- | --- | --- |
| 50 | 0.00029 | 0.00064 | 0.00172 |
| 51 | 0.00029 | 0.00064 | 0.00344 |
| 52 | **0.00029** | **0.00064** | **0.00516** |
| 53 | 0.00035 | 0.00078 | 0.00548 |
| 54 | 0.0004 | 0.00091 | 0.00581 |
| 55 | 0.00046 | 0.00105 | 0.00613 |
| 56 | 0.00051 | 0.00118 | 0.00646 |
| 57 | **0.00057** | **0.00132** | **0.00678** |
| 58 | 0.00067 | 0.0013 | 0.00674 |
| 59 | 0.00076 | 0.00129 | 0.00671 |
| 60 | 0.00086 | 0.00127 | 0.00667 |
| 61 | 0.00095 | 0.00126 | 0.00664 |
| 62 | **0.00105** | **0.00124** | **0.0066** |
| 63 | 0.00125 | 0.00146 | 0.0072 |
| 64 | 0.00144 | 0.00168 | 0.0078 |
| 65 | 0.00164 | 0.00189 | 0.00841 |
| 66 | 0.00183 | 0.00211 | 0.00901 |
| 67 | **0.00203** | **0.00233** | **0.00961** |
| 68 | 0.00241 | 0.00281 | 0.01011 |
| 69 | 0.00279 | 0.00329 | 0.01062 |
| 70 | 0.00318 | 0.00377 | 0.01112 |
| 71 | 0.00356 | 0.00425 | 0.01163 |
| 72 | **0.00394** | **0.00473** | **0.01213** |
| 73 | 0.00474 | 0.00483 | 0.01369 |
| 74 | 0.00554 | 0.00493 | 0.01525 |
| 75 | 0.00633 | 0.00503 | 0.0168 |
| 76 | 0.00713 | 0.00513 | 0.01836 |
| 77 | **0.00793** | **0.00523** | **0.01992** |
| 78 | 0.00924 | 0.00543 | 0.02161 |
| 79 | 0.01055 | 0.00563 | 0.0233 |
| 80 | 0.01185 | 0.00582 | 0.02499 |
| 81 | 0.01316 | 0.00602 | 0.02668 |
| 82 | **0.01447** | **0.00622** | **0.02837** |
| 83 | 0.01679 | 0.00717 | 0.03119 |
| 84 | 0.01911 | 0.00811 | 0.03401 |
| 85 | 0.02142 | 0.00906 | 0.03682 |
| 86 | 0.02374 | 0.01 | 0.03964 |
| 87 | **0.02606** | **0.01095** | **0.04246** |
| 88 | 0.02838 | 0.0119 | 0.04528 |
| 89 | 0.0307 | 0.01284 | 0.0481 |
| 90 | 0.03301 | 0.01379 | 0.05091 |
| 91 | 0.03533 | 0.01473 | 0.05373 |
| 92 | **0.03765** | **0.01568** | **0.05655** |
| 93 | 0.03997 | 0.01663 | 0.05937 |
| 94 | 0.04229 | 0.01757 | 0.06219 |
| 95 | 0.0446 | 0.01852 | 0.065 |
| 96 | 0.04692 | 0.01946 | 0.06782 |
| 97 | **0.04924** | **0.02041** | **0.07064** |
| 98 | 0.05156 | 0.02136 | 0.07346 |
| 99 | 0.05388 | 0.0223 | 0.07628 |
| 100 | 0.05619 | 0.02325 | 0.07909 |
