# Supplementary material for: Cost-effectiveness analysis of romosozumab for severe postmenopausal osteoporosis at very high risk of fracture in Mexico
Source: PLoS One. 2025 Feb 7;20(2):e0299673. doi: 10.1371/journal.pone.0299673 (PMC11805434; doi:10.1371/journal.pone.0299673)
Supplement: S2 Table — SE, standard error; AIC, Akaike information criterion. Parametric functions used in the model base case are shown in bold. (DOCX) [file pone.0299673.s002.docx]

**S2 Table Regression parameters fitted to FRAME trial time-to-event data**

| **Treatment** | **Parametric model** | **Intercept (SE)** | **Scale (SE)** | **Q (SE)** | **AIC** |
| --- | --- | --- | --- | --- | --- |
| **Hip fracture** |  |  |  |  |  |
| Denosumab | **Exponential** | **11.780**  **(0.258)** | **-** | **-** | **385.409** |
|  | Log logistic | 11.551  (1.297) | 0.956  (0.256) | - | 387.374 |
|  | Log normal | 14.060  (1.830) | 2.943  (0.701) | - | 386.907 |
|  | Weibull | 11.561  (1.300) | 0.958  (0.256) | - | 387.381 |
|  | Gompertz | -11.604  (0.483) | -0.001  (0.001) | - | 387.238 |
|  | Gamma | -11.532  (1.429) | 0.045  (0.259) | - | 387.379 |
| Romosozumab/denosumab | **Exponential** | **12.224**  **(0.333)** | **-** | **-** | **240.033** |
|  | Log logistic | 14.741  (2.720) | 1.443  (0.326) | - | 240.581 |
|  | Log normal | 19.184  (3.938) | 4.673  (1.423) | - | 240.388 |
|  | Weibull | 14.752  (2.724) | 1.445  (0.326) | - | 240.583 |
|  | Gompertz | -11.574  (0.554) | -0.002  (0.002) | - | 240.387 |
|  | Gamma | -14.906  (2.842) | -0.369  (0.327) | - | 240.584 |
| **Nonvertebral fracture** |  |  |  |  |  |
| Denosumab | **Exponential** | **-9.931**  **(0.103)** | **-** | **-** | **2,056.968** |
|  | Log logistic | 9.796  (0.344) | 0.965  (0.101) | - | 2,058.791 |
|  | Log normal | 11.095  (0.446) | 2.482  (0.228) | - | 2,057.494 |
|  | Weibull | 9.843  (0.349) | 0.974  (0.102) | - | 2,058.902 |
|  | Gompertz | -9.872  (0.198) | -0.000  (0.000) | - | 2,058.855 |
|  | Gamma | -9.818  (0.413) | 0.029  (0.106) | - | 2,058.892 |
|  | Generalized gamma | 11.214  (1.025) | 2.682  (0.612) | -0.108 (0.880) | 2,059.478 |
| Romosozumab/denosumab | **Exponential** | **10.118**  **(0.117)** | **-** | **-** | **1,625.266** |
|  | Log logistic | 10.048  (0.414) | 0.984  (0.113) | - | 1,627.283 |
|  | Log normal | 11.611  (0.553) | 2.644  (0.270) | - | 1,627.960 |
|  | Weibull | 10.086  (0.419) | 0.991  (0.114) | - | 1,627.259 |
|  | Gompertz | -10.162  (0.228) | 0.000  (0.001) | - | 1,627.216 |
|  | Gamma | -10.082  (0.486) | 0.009  (0.117) | - | 1,627.260 |
|  | Generalized gamma | 9.929  (1.021) | 0.776  (2.275) | 1.284 (2.998) | 1,629.241 |

SE, standard error; AIC, Akaike information criterion. Parametric functions used in the model base case are shown in bold
