# Supplementary material for: Cost-effectiveness analysis of romosozumab for severe postmenopausal osteoporosis at very high risk of fracture in Mexico
Source: PLoS One. 2025 Feb 7;20(2):e0299673. doi: 10.1371/journal.pone.0299673 (PMC11805434; doi:10.1371/journal.pone.0299673)
Supplement: S3 Table — RR, relative risk; BMD, bone mineral density. (DOCX) [file pone.0299673.s003.docx]

**S3 Table Calculation of relative risks of fracture for romosozumab versus teriparatide**

| **Input** | **Value (SE)** | **Source** |
| --- | --- | --- |
| **Change in ln(RR fracture) per % total hip BMD increase (slopes)** | | |
| Hip fracture | -0.188 (0.050) | Derived from Bouxsein et al., 2019 (39) |
| Vertebral fracture | -0.084 (0.034) | Derived from Bouxsein et al., 2019 (39) |
| Nonvertebral fracture | -0.033 (0.020) | Derived from Bouxsein et al., 2019 (39) |
| **Difference in % total hip BMD change from baseline - romosozumab versus teriparatide** | | |
| STRUCTURE trial (base case) | 3.4% (0.0032) | Langdahl et al., 2017 (19) |
| **Relative risk of fracture - romosozumab versus teriparatide** | | |
| Hip fracture | 0.75 | Calculated |
| Vertebral fracture | 0.53 | Calculated |
| Nonvertebral fracture | 0.90 | Calculated |

RR, relative risk; BMD, bone mineral density
